# Limitations of Existing Dialysis Diet Apps in Promoting User Engagement and Patient Self-Management: Quantitative Content Analysis Study

**DOI:** 10.2196/13808

**Published:** 2020-06-01

**Authors:** Jun-Hao Lim, Cordelia-Kheng-May Lim, Imliya Ibrahim, Jazlina Syahrul, Mohd Hazli Mohamed Zabil, Nor Fadhlina Zakaria, Zulfitri Azuan Mat Daud

**Affiliations:** 1 Department of Nutrition and Dietetics Faculty of Medicine and Health Sciences Universiti Putra Malaysia Serdang Malaysia; 2 Department of Computing College of Computing and Informatics Universiti Tenaga National Kajang Malaysia; 3 Department of Medicine Faculty of Medicine and Health Sciences Universiti Putra Malaysia Serdang Malaysia; 4 Research Centre of Excellence Nutrition and Non-communicable Diseases Faculty of Medicine and Health Sciences Universiti Putra Malaysia Serdang Malaysia

**Keywords:** renal apps, nutrition, dialysis, self-management, mHealth

## Abstract

**Background:**

With the unprecedented growth of mobile technology, a plethora of dialysis diet apps have been developed to promote patient dietary self-management. Nevertheless, the utility of such apps remains questionable.

**Objective:**

This study aimed to evaluate the content, features, and quality of commercial dialysis diet apps for adult dialysis patients.

**Methods:**

This study consisted of a quantitative content analysis of commercial dialysis diet apps downloaded from Google Play and the Apple App Store available in the Asian marketplace, searched for using the following keywords in English: *dialysis diet* and *diet for kidney disease*. Free and paid apps available in English that provide nutrition information for adult dialysis patients were included. Apps that were not relevant to the dialysis diet, not meant for patient self-management, or redundant were excluded. Apps were evaluated for language medium (subscore=1), credibility (subscore=1), food database (subscore=1), valuable features (subscore=12), health-behavior theory constructs (subscore=60), and technical quality (subscore=25). The relationships among the variables of interest were determined by Pearson correlation. Stepwise multiple linear regression analysis was performed to identify the features that contribute to greater technical quality of dialysis diet apps. Statistical significance was defined as *P*<.05.

**Results:**

A total of 22 out of 253 apps (8.7%) were eligible for evaluation. Based on a 100-point scale, the mean overall score of the apps was 31.30 (SD 14.28). Only 5% (1/22) of the apps offered relevant language options, and 46% (10/22) contained food databases. In addition, 54% (12/22) of the apps were not credible. The mean score for valuable features was 3.45 (SD 1.63) out of 12, in which general education (16/22, 73%), free download (15/22, 68%), and usability (13/22, 59%) were the three most popular features. However, the apps scored a mean of 13.41 (SD 11.56) out of 60 for health-behavior theory constructs. The overall app technical quality was considered poor, with a mean score of 2.70 (SD 0.41) out of 5. The scores of valuable features (*r*=.65, *P*=.001) and health-behavior theory constructs (*r*=.55, *P*=.009) were positively correlated with the overall technical quality of the commercial dialysis diet apps. Features such as free download (β=.43, *P*=.03) and usability (β=.41, *P*=.03) could significantly determine the functional quality of the apps. Health-behavior theory constructs such as self-monitoring could significantly predict both the subjective quality (β=.55, *P*=.008) and the engagement quality (β=.66, *P*=.001) of the apps, whereas the information quality domain could be determined by plan or orders (β=.48, *P*=.007) and knowledge (β=.45, *P*=.01).

**Conclusions:**

Although most of the available commercial dialysis diet apps are free and easy to use, they are subject to theory deficiency, limited language options, and a lack of food databases, credibility, tailored education, and overall technical quality.

## Introduction

With the unprecedented growth of mobile technology, the use of mobile phones is ubiquitous around the globe. Such usage has been proliferating over the years, with a world penetration rate of 67% in 2019 [1]. In this modern era, mobile phones are not only communication tools but also indispensable devices that enable users to perform a variety of activities, such as those related to entertainment, social media, fitness, and health care.

Recently, there has been growing interest in mobile health (mHealth) app development. According to a survey [2], approximately 58,000 mHealth app publishers existed in 2016. Among various categories, nutrition-related apps were found to be the most downloaded mHealth apps [3]. The positive effects of nutrition-related mHealth apps as self-monitoring tools in managing chronic diseases, particularly concerning weight management, have been supported by a recent systematic review and meta-analysis [4]. Moreover, the same study has also addressed a gap pertaining to the effects of dietary mHealth apps on chronic kidney diseases (CKDs).

Diet modification is one of the most crucial components of comprehensive dialysis treatment [5]. Poor dietary adherence will result in life-threatening complications [6-8]. However, diet modification for dialysis patients is challenging due to the complexity of the renal diet. Such diet modification requires a substantial amount of patient self-management skills to integrate and implement the complex dietary recommendations over the course of one’s lifetime [9].

A plethora of dialysis diet apps are now available in mobile app stores. Their roles as dietary self-management tools (ie, diet trackers, food diaries, calorie counting functions, and nutrition recommendations) for dialysis patients as adjuncts to dietetics counseling have been increasingly advocated [10,11]. However, there is no strong conclusive evidence to support the clinical efficacy of these apps [12]. Poor app engagement and usability issues are believed to be the reasons for the limited utility of these apps [11]. In addition, due to the absence of consensus standards and development guidelines for mHealth apps [13], the quality of commercial dialysis diet apps is questionable.

An earlier study found that approximately half of the commercial diet apps for kidney diseases were not credible and only had fair technical quality [14]. Nevertheless, other important aspects of commercial dialysis diet apps that may be linked to greater user acceptance, engagement, and effectiveness have yet to be explored, such as health behavioral theory [15], a set of valuable features [16], and other aspects, such as language medium [17] and food databases [18].

Therefore, this study aims to examine the content, features, and quality of commercial dialysis diet apps to inform health care professionals and patients about the current state of dialysis diet apps and to address the aspects of apps that must be improved.

## Methods

### Study Design

In this study, we performed a quantitative content analysis of commercial dialysis diet apps from the two most popular mobile platforms in the Asian marketplace: Google Android (ie, downloaded from the Google Play store) and Apple iOS (ie, downloaded from the Apple App Store). The content, features, and quality of the eligible apps were evaluated and quantified using a predefined scoring system. Considering the nature of the study design (ie, desk-based study), ethical approval was exempted from this study.

### Sampling Method

Dialysis diet apps were sampled from Google Play and the Apple App Store for the Asian marketplace using plausible keywords that dialysis patients would use to search for renal diet apps (ie, *dialysis diet* and *diet for kidney disease*). The search was conducted by two research staff members from September 26, 2018, to October 31, 2018. The apps identified in the initial search were screened for eligibility using predefined selection criteria. The inclusion criteria for apps in this study were as follows: apps were free or paid, were available in English, and provided nutrition information, including fluid control for adult dialysis patients. In contrast, apps were excluded if they were not relevant to the dialysis diet, not meant for patient self-management, or redundant. Screening was conducted independently by two study staff members to minimize researcher bias. Discrepancies in the screening results were compared and discussed between the staff members before a final list of dialysis diet apps was constructed for further evaluation.

### App Evaluation

Eligible dialysis diet apps were evaluated concerning six main aspects. These included the following: (1) the presence of valuable features, (2) the extent of health-behavior theory incorporation, (3) credibility, (4) technical quality, (5) the option of a language medium, and (6) the presence of a food database. The evaluation aspects included in this study were chosen based on the extant literature pertaining to the features and characteristics of mHealth apps that might be associated with greater user acceptance, engagement, and effectiveness [14-18].

### Scoring System

#### Overview

A scoring system encompassing all evaluation aspects was developed to quantify the evaluation outcomes. The scoring system consisted of a rubric and scale adopted from the literature [15,16,19]. Apps were scored if they fulfilled the predetermined criteria according to the scoring distribution: language medium (subscore=1), credibility (subscore=1), food databases (subscore=1), valuable features (subscore=12), health-behavior theory constructs (subscore=60), and technical quality (subscore=25). The scoring distribution was constructed as the number of components within each criterion and/or their respective scoring scale. For instance, the subscore assigned to the aspect of valuable features was 12 because it consists of 12 evaluation features [16], as shown in Multimedia Appendix 1. Health-behavior theory [15] and technical quality [19] were scored according to their respective scales as described above. Since credibility, language, and food databases are stand-alone features, they only contributed a score of 1 each to the overall score. The overall score of each app ranged from 0 to 100, with a higher score indicating higher quality in terms of content and features that were thought to be linked to a greater acceptance, engagement, and effectiveness of the mHealth app.

#### Valuable Features

Eligible apps were assessed for the presence of valuable features adopted from the previous content analysis of mHealth apps [16]. There were 12 valuable features found to be associated with user engagement and positive user rating [16]: (1) export of data, (2) gamification, (3) general education, (4) plan or orders, (5) reminders, (6) community forum, (7) social media, (8) addressing of symptoms, (9) tailored education, (10) tracker, (11) cost (ie, free download), and (12) usability. The description of each feature is presented in Multimedia Appendix 1. Using a binary system, apps were given a score of 1 to indicate the presence of a specific feature. Otherwise, a score of 0 was given. Eventually, the score of each feature was summed, with the overall subscore ranging from 0 to 12.

#### Health-Behavior Theory

A rubric utilized by previous studies [15,20] was adopted to examine the extent of incorporating health-behavior theory constructs into commercial dialysis diet apps. Eligible apps were assessed for the presence of 12 theoretical constructs (ie, knowledge, perceived benefits, perceived barriers, perceived risks, self-efficacy, social norm, self-monitoring, goal setting, stimulus control, self-reward, social support, and vicarious learning), grounded from the four most commonly used health-behavior theories in health apps, including (1) the health belief model, (2) the transtheoretical model, (3) the theory of planned behavior, and (4) social cognitive theory. Then, each of the constructs was coded based on six levels of user interaction. The construct was rated as 0 if user interaction was absent, 1 if it involved general information, 2 for assessment, 3 for feedback, 4 for general assistance, and 5 for individually tailored assistance (see Multimedia Appendix 2). The subscores for this section ranged from 0 to 60 (ie, 12 constructs × six levels of user interaction), with a higher score indicating a greater extent of the incorporation of health-behavior theory.

#### Credibility

The content of the eligible apps was examined by comparing it with the dietary recommendations for the adult CKD population [21], which were derived from numerous nutrition guidelines [22-26]. Apps with inconsistent information or without reliable references were labeled as not credible.

Conversely, for apps that function solely as diet trackers, their credibility was assessed primarily through the accuracy of their food databases. A total of 20 food items (ie, four food items per major food group: cereals, protein, vegetables, fruits, and fat), which represent an average number of food items consumed by a person per day [27], were randomly selected for comparison using cross-classification analysis, which is a widely used method to assess agreement in validation studies involving food nutrients [28,29]. Apps that showed more than 7% gross misclassification [30] were considered not credible. In the absence of a gold standard, the nutrient content of food in the databases of the dialysis diet apps was compared with that of the computer software Nutritionist Pro, version 2.2.16 (First DataBank Inc) [31]. Nutritionist Pro has been widely utilized to assess dietary intake in many published research studies [32-34], including for CKD [35-37]. In addition, it has also been used to analyze dietary data in numerous validation studies on dietary assessment tools, such as food frequency questionnaires [38,39]. Nutritionist Pro contains food databases of various regions, such as the United States Department of Agriculture, the Canadian Nutrient File, and UK, European, and Malaysian food databases. As the only food database for Asian countries in Nutritionist Pro, the Malaysian food database was used in this study to determine the credibility of the apps.

In this study, apps that provided inaccurate or partially accurate information or were not from reliable sources were given a score of 0. In contrast, a score of 1 was given to credible apps.

#### Technical Quality

The technical quality of the apps was evaluated using the Mobile Application Rating Scale (MARS) [19], which is a validated tool specifically designed to evaluate the technical quality of mHealth apps based on five quality domains: (1) engagement (ie, entertainment, interest, customization, interactivity, and fit to target group), (2) functionality (ie, performance, ease of use, navigation, and gestural design), (3) aesthetics (ie, layout, graphic design, and visual appeal), (4) information (ie, quality and quantity of visual information, credibility, goal, and accuracy of app description), and (5) subjective quality (ie, recommendation, willingness to pay, willingness to use in future, and overall satisfaction). In this study, the MARS has been adopted without any modification to its items and domains to evaluate the technical quality of the apps. However, to fulfill the needs for this study, the scoring method has been adapted by summing the subscores of all domains. Each question was scored on a scale of 1 (inadequate) to 5 (excellent), with the total score ranging from 5 (minimum) to 25 (maximum). The validity and reliability of the MARS with the revised scoring method has been determined using the evaluated apps. Good interrater reliability (intraclass correlation coefficient [ICC]=.813) and internal consistency (Cronbach alpha=.751-.874) were found. The construct validity of the MARS was determined using convergent validity with an average variance extracted of 0.683-0.813 and composite reliability of 0.771-0.893 across domains.

#### Language Medium

The language barrier was identified as a limiting factor that affects mHealth app accessibility and effectiveness [17]. Implications of the language barrier are believed to be more profound for users from countries where English is not the first language (ie, Asian countries). For instance, a study showed that many patients in Asian countries have limited English proficiency [40]. Since the dialysis diet apps in this study were sampled from app stores for the Asian marketplace, the language medium was also examined in this study. An app was given a score of 1 if a language option relevant to the Asian marketplace was available. Otherwise, a score of 0 was given.

#### Food Database

Food databases are one of the most common features in nutritional apps [18] and serve as an essential component for diet tracking that would be needed for dietary self-management. Diet trackers have been found to be a core feature in CKD apps being trialed or tested in the literature [11]. In this study, apps were given a score of 1 if food databases were found in the apps and 0 if food databases were absent, regardless of app credibility.

### Raters

Eligible apps were rated by two trained study staff members with dietetics backgrounds. Prior to the evaluation, training was conducted to explain and discuss the scoring scheme with the raters. Then, three non-renal-related diet apps were trialed using the scoring scheme. Rater agreement was determined using the kappa statistic for categorical data and the ICC for continuous data. Good interrater reliability was determined, with kappa and ICC values equal to .798 and .762, respectively. Discrepancies in the rating were discussed until a consensus was reached. Then, each eligible app was downloaded and coded independently by the raters for approximately 1 week. The final score of each app was obtained by averaging the scores from the raters.

### Statistical Analysis

Descriptive statistics were used to summarize the findings of this study. All data were analyzed using SPSS, version 25.0 (IBM Corp). Categorical variables were expressed as frequencies and percentages, while continuous data were expressed as the mean (SD). The relationships among variables of interest were determined by Pearson correlation. Stepwise multiple linear regression analysis was performed to identify the features that predict the technical quality of apps. Statistical significance was defined as *P*<.05.

## Results

### Search Results

**Figure 1:**
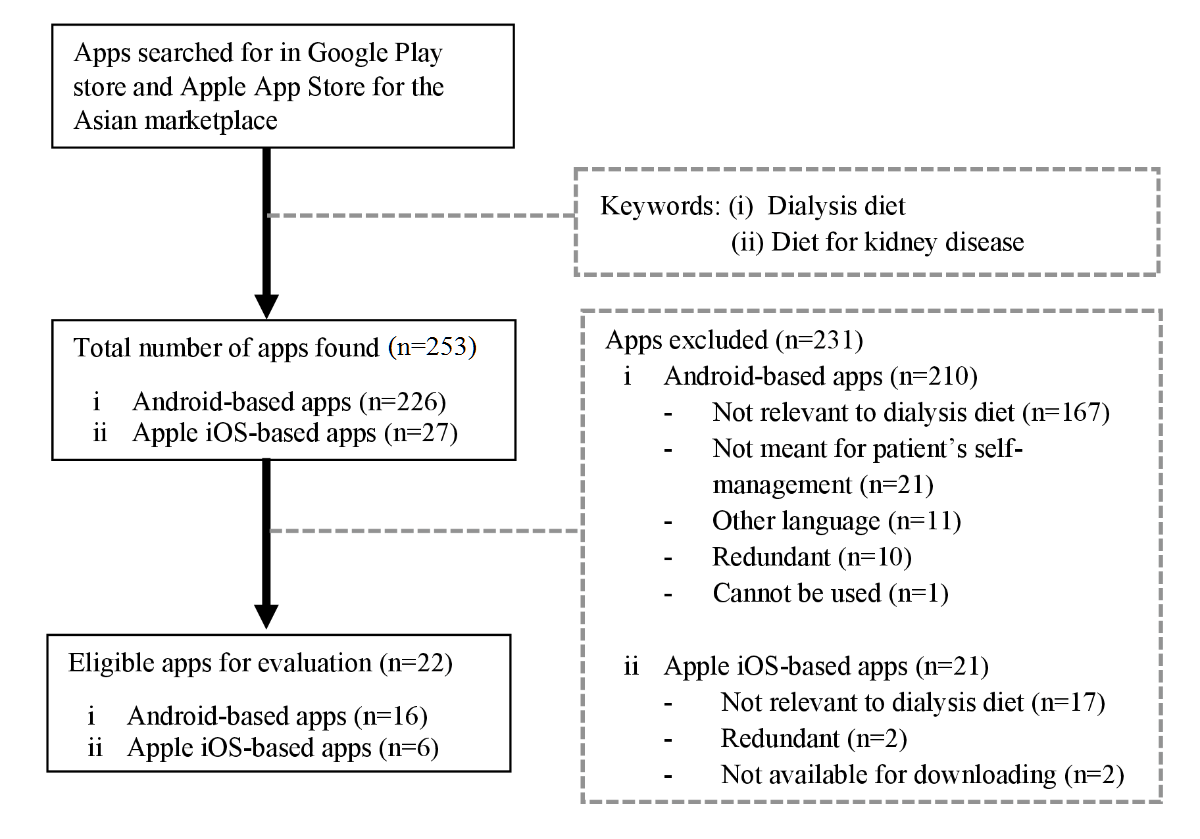
Flow diagram of the selection process and filtering results for the content analysis of existing dialysis-specific diet apps.

A total of 253 apps were identified in the initial search. However, only 8.7% (22/253) of the apps were eligible for evaluation. Of these apps, 73% (16/22) were Android-based apps, while the remaining 27% (6/22) were Apple iOS apps. A large proportion of the apps (231/253, 91.3%) were excluded, as they were not relevant to dialysis diets, such as non-dialysis-specific diet apps (ie, fitness, diabetes, uric acid, kidney stones, etc) and calculators (ie, glomerular filtration rate calculator), which did not provide any dialysis-related diet information and were not meant for patient self-management (ie, journals, medical pocketbooks, etc). Other reasons for exclusion were redundant apps, apps available only in other languages (ie, Urdu, German, and Spanish), and apps that could not be downloaded or used. The sampling details are presented in Figure 1.

### Evaluation Outcomes

Table 1 depicts the summary scores of the app evaluation. Based on the 100-point scale, the mean overall app score was 31.30 (SD 14.28), ranging from 10.28 (lowest) to 53.82 (highest). However, most of the apps (19/22, 86%) scored less than 50 points. The mean score acquired by Android-based apps was 35.13 (SD 13.18), while Apple iOS-based apps obtained an average score of 21.09 (SD 12.79). The scores of the evaluated apps are presented in Multimedia Appendix 3.

Only 5% (1/22) of the apps offered language options relevant to the Asian marketplace, while approximately 46% (10/22) contained food databases (see Table 1). In addition, 54% (12/22) of the evaluated apps were not credible, and Android-based apps (9/16, 56%) were more credible than Apple iOS-based apps (1/6, 17%). Commercial dialysis diet apps scored 3.45 (SD 1.63) out of 12 for valuable features; Android-based dialysis diet apps (3.88, SD 1.59) contained more valuable features than did Apple iOS-based dialysis diet apps (2.33, SD 1.21), as shown in Table 1.

The mean overall MARS score of commercial dialysis diet apps was 13.48 (SD 2.05) out of a total score of 25 (see Table 1). Android dialysis diet apps (14.04, SD 1.87) have better technical quality than Apple iOS apps (12.01, SD 1.92). The mean score of commercial dialysis diet apps across the five MARS quality domains was 2.70 (SD 0.41) out of 5, with the highest score being for functionality (3.79, SD 0.45), followed by those for aesthetics (2.95, SD 0.44), engagement (2.40, SD 0.66), information (2.27, SD 0.59), and subject quality (2.08, SD 0.48).

The presence of valuable features in commercial dialysis diet apps is depicted in Multimedia Appendix 4. The app that contained the highest number of valuable features (ie, seven) was the Android-based app *Renal Care Compass*. In contrast, the Apple iOS app *Healthy Kidneys Grocery List* had the least valuable features (ie, one). The three most popular valuable features found in commercial dialysis diet apps were general education (16/22, 73%), followed by free download (15/22, 68%) and usability (13/22, 59%). Moreover, features such as gamification (1/22, 5%), tailored education (1/22, 5%), social media (0/22, 0%), and community forums (0/22, 0%) were the least incorporated features in commercial dialysis diet apps.

**Figure 2:**
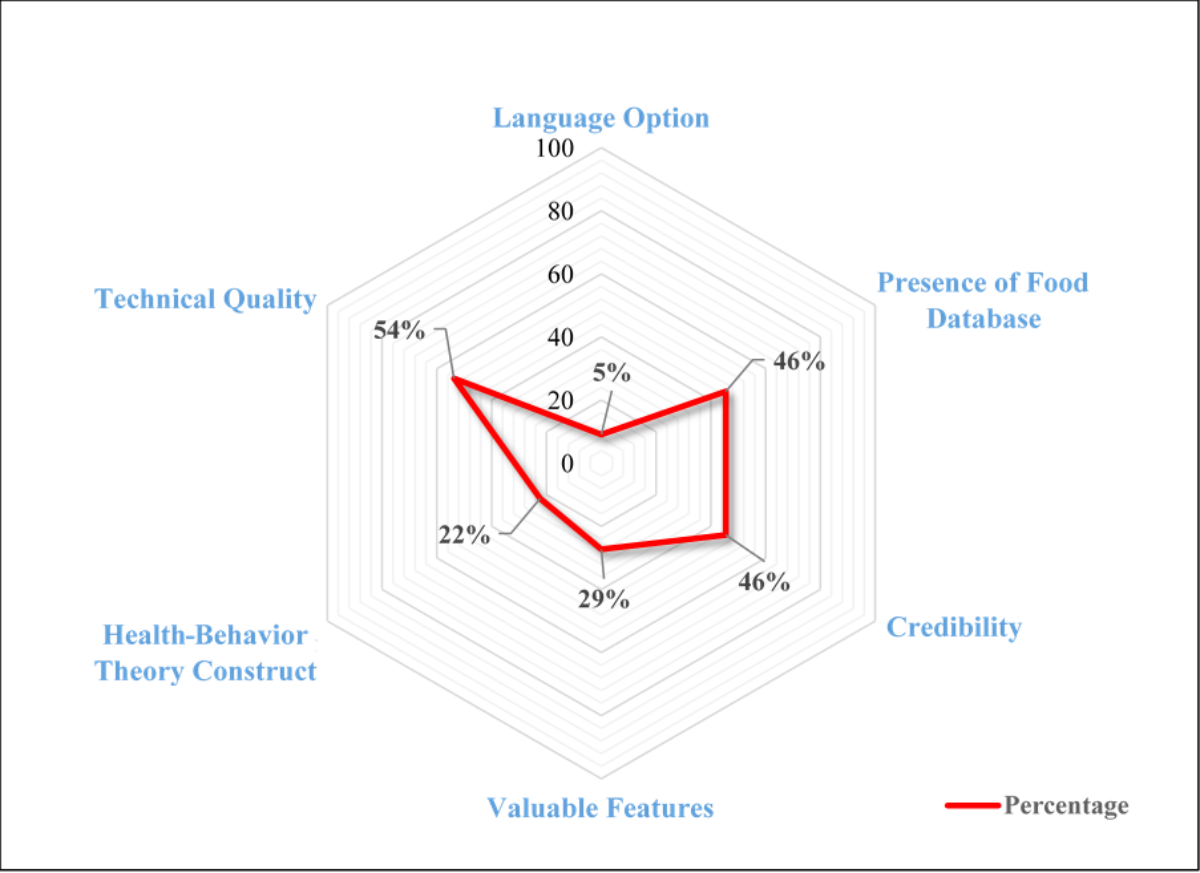
Radar chart for the evaluation results of existing dialysis-specific diet apps.

Out of a total score of 60, the mean score of commercial dialysis diet apps for health-behavior theory constructs was 13.41 (SD 11.56) (see Table 1). In general, Android-based dialysis diet apps (16.16, SD 11.11) applied theoretical constructs to a greater extent than did Apple iOS-based dialysis diet apps (6.08, SD 10.10). The health-behavior theory constructs integrated into commercial dialysis diet apps are presented in Multimedia Appendix 5. The Android-based dialysis diet app *Renal Disease Kidney Diet Tips Symptoms & Foods* was the most theory-based dialysis diet app. Surprisingly, 4 out of 6 (67%) Apple iOS-based dialysis diet apps did not integrate any theoretical construct evaluated in this study. Knowledge (17/22, 77%), goal setting (15/22, 68%), and self-efficacy (13/22, 59%) were the most widely used theoretical constructs in commercial dialysis diet apps. In contrast, perceived barrier (2/22, 9%), self-reward (2/22, 9%), social support (1/22, 5%), and vicarious learning (1/22, 5%) were the least incorporated theoretical constructs. Collectively, the findings of the evaluation of commercial dialysis diet apps by evaluating different aspects are summarized in percentages and illustrated in Figure 2.

Table 2 presents the relationships among the valuable features, health-behavior theory, and technical quality of commercial dialysis diet apps. Except for aesthetics and functionality quality domains, valuable features were significantly correlated with overall technical quality (*r*=.65, *P*=.001), the engagement quality domain (*r*=.60, *P*=.003), the information quality domain (*r*=.61, *P*=.002), and the subjective quality domain (*r*=.61, *P*=.003). Similarly, health-behavior theory was significantly correlated with overall technical quality (*r*=.55, *P*=.009), the engagement quality domain (*r*=.45, *P*=.04), and the information quality domain (*r*=.53, *P*=.01), but not with the aesthetics, functionality, and subjective quality domains (*P*>.05).

Stepwise regression analysis indicated that only cost (β=.49, *P*=.005) and self-monitoring (β=.46, *P*=.009) could significantly predict the overall quality of commercial dialysis diet apps (see Table 3). In addition, self-monitoring was also a predictor of the engagement (β=.66, *P*=.001) and subjective quality (β=.55, *P*=.008) domains. The functionality quality of the app could be determined by cost (β=.43, *P*=.03) and usability (β=.41, *P*=.03), while the information quality domain could be determined by plan or orders (β=.48, *P*=.007) and knowledge (β=.45, *P*=.01).

**Table 1:** Summary scores of the evaluated dialysis diet apps.

Evaluating aspects		Overall (N=22)	Android-based apps (n=16)		Apple iOS-based apps (n=6)	
**Language (score out of 1)**						
	Total score, mean (SD)	0.05 (0.21)	N/A^a^	N/A		
	English only, n (%)	21 (95)	15 (94)	6 (100)		
	Multilanguage, n (%)	1 (5)	1 (6)	0 (0)		
**Food database (score out of 1)**						
	Total score, mean (SD)	0.45 (0.51)	N/A	N/A		
	Present, n (%)	10 (46)	7 (44)	3 (50)		
	Absent, n (%)	12 (54)	9 (56)	3 (50)		
**Credibility (score out of 1)**						
	Total score, mean (SD)	0.45 (0.51)	N/A	N/A		
	Yes, n (%)	10 (46)	9 (56)	1 (17)		
	No, n (%)	12 (54)	7 (44)	5 (83)		
Valuable features (score out of 12), mean (SD)		3.45 (1.63)	3.88 (1.59)		2.33 (1.21)	
Theory constructs (score out of 60), mean (SD)		13.41 (11.56)	16.16 (11.11)		6.08 (10.10)	
**Technical quality (score out of 25), mean (SD)**						
	Total score	13.48 (2.05)	14.04 (1.87)	12.01 (1.92)		
	Engagement	2.40 (0.66)	2.60 (0.60 )	1.88 (0.55)		
	Functionality	3.79 (0.45)	3.81 (0.50)	3.73 (0.33)		
	Aesthetics	2.95 (0.44)	3.03 (0.43)	2.72 (0.39)		
	Information	2.27 (0.59)	2.43 (0.52)	1.83 (0.60)		
	Subjective quality	2.08 (0.48)	2.17 (0.50)	1.84 (0.32)		
	Mobile Application Rating Scale score	2.70 (0.41)	2.81 (0.37)	2.40 (0.38)		
Total score (score out of 100), mean (SD)		31.30 (14.28)	35.13 (13.18)		21.09 (12.79)	

^a^N/A: not applicable.

**Table 2:** Correlations^a^ between mean scores of technical quality with mean scores of valuable features and health-behavior theory for evaluated dialysis diet apps (N=22).

Technical quality	Valuable features		Health-behavior theory	
	*r*	*P* value	*r*	*P* value
Overall	.65	.001	.55	.009
Engagement	.60	.003	.45	.04
Functionality	.27	.22	.39	.07
Aesthetics	.36	.10	.37	.09
Information	.61	.002	.53	.01
Subjective quality	.61	.003	.36	.10

^a^Analyzed using Pearson correlation.

**Table 3:** Predictors of technical quality in evaluated dialysis diet apps (N=22)^a^.

Technical quality and predictors		R^2^		B		SE	β		*t* test		*P* value	
**Overall quality**												
	Self-monitoring		.56		1.85	0.64		.46		2.90		.009
	Cost		N/A^b^		2.14	0.68		.49		3.14		.005
**Engagement**												
	Self-monitoring		.43		0.85	0.22		.66		3.92		.001
**Functionality**												
	Cost		.44		0.41	0.17		.43		2.42		.03
	Usability		N/A		0.37	0.16		.41		2.33		.03
**Information**												
	Plan or orders		.54		0.72	0.24		.48		3.00		.007
	Knowledge		N/A		0.62	0.22		.45		2.90		.01
**Subjective quality**												
	Self-monitoring		.31		0.52	0.18		.55		2.97		.008

^a^Analyzed using stepwise multiple linear regression.

^b^N/A: not applicable.

## Discussion

### Principal Findings

Based on the findings of this study, only a limited number of commercial renal diet apps (22/253, 8.7%) are available for dialysis patients. Moreover, these apps were found to be lacking in language options relevant to Asian marketplaces (1/22, 5%) and food databases (10/22, 46%). They also have poor technical quality (mean 13.48, SD 2.05, out of 25) associated with limited valuable features (mean 3.50, SD 1.68, out of 12) and health-behavior theory incorporation (mean 13.41, SD 11.56, out of 60).

Renal patients have shown a growing interest in using mHealth apps [41]. Unfortunately, despite having numerous renal diet apps in mobile app stores, only a limited number of apps are likely to fulfill the needs of dialysis patients, regardless of their quality. In addition, renal apps that are meant for patient self-management are still limited [42]. Choices of dialysis diet apps are further limited by the absence of language options, as not all patients are literate in the English language, especially in most Asian countries [40]. In this study, only one app (ie, *Aqualert Drink Water Tracker & Reminder Google Fit*) offered language options relevant to Asian marketplaces, including Mandarin, Thai, Indonesian, Korean, and Japanese languages. Moreover, although language options (ie, Catalan and Spanish) were found in the app *Pukono*, these languages are not relevant to the Asian marketplace. The implications of the possible language barrier in mHealth apps have been discussed in a previous study [43]. The study showed that most mobile phone users prefer to use apps in their primary language [44], which can lead to greater user engagement and prevent the misinterpretation of health information.

This study found that the overall technical quality of commercial dialysis diet apps assessed by the MARS was poor. Functionality was found to be the top-rated quality domain of the MARS, associated with the usability of apps. Generally, commercial dialysis diet apps work well with minimal technical errors. However, serious usability problems, such as lagging, were detected in certain apps (ie, *Chronic Kidney Disease*), which may cause frustration among their users [45]. In addition to app performance, functionality also refers to user experience. Both dietitians and patients prefer an app that is simple and intuitive to use [46,47]. Although the majority of the commercial dialysis diet apps (13/22, 59%) were rated as *easy to use*, features that allow for easy control interactions were absent in certain apps. For instance, users must fill in their basic information (ie, age, gender, and height) whenever logging in to the apps. In addition, important gestural designs, such as pinch for zooming, were also absent in certain apps (ie, *Renal TRKRR*). Considering the possible vision problems in dialysis patients secondary to aging or concomitant disease (ie, diabetic retinopathy), the content of the apps might be too small to be seen.

A low retention rate remains a critical issue with mHealth apps [48]. Commercial dialysis diet apps are lacking in interactive features (ie, feedback), making the apps less engaging to users. Although gamification is a trending feature for promoting user engagement [49], it is one of the least exploited features in commercial dialysis diet apps. Based on our findings, the engagement quality of apps assessed by the MARS can be improved by incorporating self-monitoring features, as they promote user interaction (ie, diet tracking and feedback) [50]. Although 46% (10/22) of commercial dialysis diet apps offer self-monitoring features, the food items available in the database are mainly Western foods. This might limit their usefulness, especially for users from non-Western countries. A local food database is necessary to provide accurate dietary self-monitoring [51]. Dietary assessment using a foreign food database may cause a significant error during dietary assessment [14].

As expected, the evaluated apps scored poorly on the information quality domain of the MARS due to the lack of accuracy in the health information provided. Credibility is a prerequisite for useful mHealth apps, and it is always the greatest concern of health care professionals [46]. More than half of the commercial dialysis diet apps evaluated in this study were not credible, which may create uncertainties among health care professionals in recommending mHealth apps to their patients. From the patient’s perspective, unreliable health information in the apps can bring serious detrimental effects, exacerbating the health of this vulnerable population [10]. The lack of input from health care professionals was regarded as the main reason for misinformation in commercial mHealth apps [52]. Thus, the involvement of health care professionals (ie, renal dietitians and nephrologists) in mHealth app development is advocated to ensure the credibility of the health information given [14]. Furthermore, dialysis diet apps tend to have better information quality if they provide information and guidance about renal diet (ie, dietary plan and knowledge) compared to those that function solely as a tracker (ie, *Renal TRKRR*). Moreover, none of the evaluated diet apps in this study were previously tested in a clinical trial. Although a previous study found potential clinical benefits of renal diet apps in the dialysis population [12] (ie, *BalanceLog* and *Dietary Intake Monitoring Application [DIMA]*), they were not available in the mobile app stores of Google and Apple during the evaluation period.

The aesthetic quality domains of the evaluated apps in this study were below average. Of these, the color, design, and layout of commercial dialysis diet apps need to be improved. Although color is not a primary concern when designing an app, it exerts a profound effect on user experience and overall satisfaction [53]. Poor color combination, especially the background color, may affect the readability of the text [54].

Moreover, the evaluated apps scored the lowest for the domain of subjective quality in the MARS. Approximately 46% (10/22) of the apps were rated *below average*, and minimal usage (ie, less than two times per year) was expected for most apps (18/22, 82%). The raters involved in this study represent the perspective of dietitians on commercial dialysis diet apps. Out of 22 apps, only 7 (36%) were likely to be recommended to dialysis patients. In addition, we found that dietitians (ie, raters) prefer apps with self-monitoring features that allow dialysis patients to monitor their nutrient intake.

The cost of apps is also an important criterion to be considered. Although paid apps are always deemed to be superior to free apps [16], it is not the sole indicator of better quality for commercial dialysis diet apps. In this study, we found that free dialysis diet apps outperformed paid apps in almost every evaluated aspect. The possible reason is that rather than offering additional features and functions, paid apps are generally meant for better user experience (ie, ad free) [16]. This is supported by the features offered by in-app purchases. Only a small subset (2/22, 9%) of the evaluated apps offer in-app purchases in this study. However, they are mainly used to avoid advertisements and do not contribute to any additional features evaluated. This may also explain the finding that Android apps outperform iOS apps, as most of the iOS dialysis diet apps (5/6, 83%) are paid apps compared to Android dialysis diet apps (2/16, 13%).

Health-behavior theory plays a crucial role in mHealth apps [55,56]. Similar to previous studies [15,57], the constructs of social cognitive theory (ie, knowledge, goal setting, and self-efficacy) were the most common theoretical constructs found in commercial dialysis diet apps. They have been used in designing mHealth app interventions for chronic diseases, including hemodialysis populations [58]. Consistent with previous findings [15], the extent of theory application in commercial dialysis diet apps was restricted to general information and general assistance, which are considered insufficient to bring about significant and long-term behavior change [59]. Instead, individualized dietary feedback based on assessments is more likely to promote sustainable behavior changes [60]. In this study, only one app (ie, *Renal Disease Kidney Diet Tips Symptoms & Foods*) was found to provide individualized assistance through social support (ie, online consultation).

### Strengths and Limitations

Since Google Android and Apple iOS are the most popular mobile platforms worldwide, the findings of this study can serve as a reference for global health care professionals and the dialysis population. However, this study has several limitations. The commercial dialysis diet apps included in this study were searched for over a short period (ie, September 26 to October 31, 2018) using English keywords only. In addition, they were also confined to the apps available for Google Android and Apple iOS for the Asian marketplace only. Thus, the findings cannot be inferred for apps launched after the study period and those available in other platforms and languages.

### Comparison With Prior Work

Prior studies had been conducted to evaluate commercial renal apps designed for both renal patients and health care professionals. These include studies pertaining to diet apps for general kidney diseases [14] and health apps specific to CKD management [42,61]. In comparison, this study was focused on diet apps designed specifically for dietary self-management in the dialysis population. In addition, the use of different mobile platforms as well as the keywords used to identify apps resulted in a different number of renal apps being evaluated. Moreover, this study evaluated apps on different aspects compared to previous studies, which focused more on the functionality and content of the apps rather than their language medium, food database, presence of valuable user features, and incorporation of health-behavior theory.

Despite different app pools, our findings were consistent with a previous study in which 45.5% of renal diet apps were found to be not credible [14]. Similarly, functionality was found to be the top technical quality domain assessed by the MARS. In contrast, the overall mean technical quality of dialysis-specific diet apps in this study was slightly lower than that of the previous study [14].

In addition, the findings of this study are also consistent with those of a previous study [42] in which limited apps were found to be available for patient dietary self-management (8.7% vs 9.0%). Despite different evaluation aspects, we agreed with the study conducted by Lee et al [61], which found that commercial renal apps had limited capability to support renal patient self-management.

### Conclusions

Although most of the available commercial dialysis diet apps are free and easy to use, they are subject to a possible language barrier, theory deficiency, and a lack of credibility, food databases, and tailored education. Thus, they might have limited potential to promote user engagement and patient dietary self-management. Further research efforts are needed to develop a theory- and evidence-based dialysis diet app equipped with desirable features to promote dietary self-management in the dialysis population.

## Appendix

Multimedia Appendix 1 The valuable features with descriptions adopted from Mendiola et al, 2015.

Multimedia Appendix 2 The definitions of user interaction for health-behavior theory adopted from Davis et al, 2016.

Multimedia Appendix 3 Scores of the evaluated dialysis diet apps (N=22).

Multimedia Appendix 4 The presence of valuable features in evaluated dialysis diet apps from Google Play and the Apple App Store (N=22).

Multimedia Appendix 5 The presence of health-behavior theory constructs in evaluated renal diet apps for dialysis from Google Play and the Apple App Store (N=22).

## Abbreviations

AJE: American Journal Experts
CKD: chronic kidney disease
DIMA: Dietary Intake Monitoring Application
ICC: intraclass correlation coefficient
MARS: Mobile Application Rating Scale
mHealth: mobile health
