# Supplementary material for: Limitations of Existing Dialysis Diet Apps in Promoting User Engagement and Patient Self-Management: Quantitative Content Analysis Study
Source: JMIR Mhealth Uhealth. 2020 Jun 1;8(6):e13808. doi: 10.2196/13808 (PMC7296424; doi:10.2196/13808)
Supplement: Multimedia Appendix 1 [file mhealth_v8i6e13808_app1.docx]

| **App feature or characteristic** | **Description of feature or characteristic** |
| --- | --- |
| 1. Export of Data | - Feature that allows the user to communicate or send information/ data to a health care provider (eg, email and EHR/PHR) |
| 1. Gamification | - Feature that offers points, badges, or movement through levels as a health objective is achieved or the more a patient is engage |
| 1. General education | - Feature that provides basic educational material about a disease/condition, including causes, treatment, or management |
| 1. Plan or orders | - Feature that provides a plan of action for reaching target goal, including specific, executable steps to guide the process |
| 1. Reminder | - Feature that prompts the user to partake in a specific behavior through the use of a predetermined alert |
| 1. Community Forum | - Feature that functions as a message board or chat room and allows likeminded individuals, whether patients with similar health conditions or their caregivers, the opportunity to share questions and experiences |
| 1. Social media | - Feature that connects the user to Facebook, Twitter, or other social media platforms, thereby allowing the user to commu- nicate progress with family, friends, colleagues, or others with ties to the user |
| 1. Addresses symptoms | - Feature that addresses and assists in managing a disease that is associated with pain or other noticeable symptom(s) |
| 1. Tailored Education | - Feature that offers patient-specific education tailored to a person’s needs, interests and usage depending on his/her stage or progression of disease (eg, week of pregnancy) |
| 1. Tracker | - Feature that allows for self-monitoring by recording informa- tion in order to modify personal attitudes or behaviors to achieve a predetermined goal or outcome |
| 1. Cost | - Identification of cost of the app (free, upfront payment, and/or in-app purchases) |
| 1. Usability | - Identification of satisfactory usability based on compliance with five interface design heuristics |

Multimedia Appendix 1: The Valuable Features with Descriptions Adopted from Mendiola et al, 2015.
