# Supplementary material for: Limitations of Existing Dialysis Diet Apps in Promoting User Engagement and Patient Self-Management: Quantitative Content Analysis Study
Source: JMIR Mhealth Uhealth. 2020 Jun 1;8(6):e13808. doi: 10.2196/13808 (PMC7296424; doi:10.2196/13808)
Supplement: Multimedia Appendix 2 [file mhealth_v8i6e13808_app2.docx]

Multimedia Appendix 2: The Definitions of User Interaction for Health Behavior Theory Adopted from Davis et al, 2016.

| **Level** | **Category of Interaction** | **Description** |
| --- | --- | --- |
| 1 | Information or guidelines | App provides primarily general information or data that are not individualized |
| 2 | Assessment | App asks the user for current behavioural practices or use of strategies |
| 3 | Feedback | App comments on the user’s current behavioural practices or strategies as supplied in Item 2 |
| 4 | General assistance | App offers non-individualized suggestions about how to change or apply a strategy that are not responses to any assessment (Items 2) and do not require feedback (Items 3) |
| 5 | Individually tailored assistance | App has suggestions about how to change or apply a strategy specifically tailored to the user |
