# Supplementary material for: Limitations of Existing Dialysis Diet Apps in Promoting User Engagement and Patient Self-Management: Quantitative Content Analysis Study
Source: JMIR Mhealth Uhealth. 2020 Jun 1;8(6):e13808. doi: 10.2196/13808 (PMC7296424; doi:10.2196/13808)
Supplement: Multimedia Appendix 3 [file mhealth_v8i6e13808_app3.docx]

| **Dialysis Diet Apps** | **Version** | **Last updated** | **Evaluating Aspects (Scores)** | | | | | | **Total Score** |
| --- | --- | --- | --- | --- | --- | --- | --- | --- | --- |
|  |  |  | **Language**  **Option** | **Food Database** | **Credibility** | **Valuable Features** | **Health Behavior Theory** | **Technical Quality** |  |
| **Android-Based** | | | | | | | | | |
| 1. Phosphorus Foods Diet Guide | 1.0 | December 14, 2017 | 0 | 1 | 1 | 4 | 20.5 | 13.63 | 40.13 |
| 2. Renal Disease Kidney Diet Tips  Symptoms & Foods | 2.3 | May 14, 2018 | 0 | 0 | 0 | 5 | 34.0 | 13.22 | 52.22 |
| 3. Prevent Kidney Disease | 1.0 | August 16, 2017 | 0 | 0 | 0 | 4 | 24.0 | 11.30 | 39.30 |
| 4. CKD (Chronic Kidney Disease) | 1.0 | September 23, 2016 | 0 | 0 | 0 | 1 | 1.0 | 12.07 | 14.07 |
| 5. Kidney Friend | 1.1 | N/A | 0 | 0 | 1 | 5 | 26.5 | 13.19 | 45.69 |
| 6. Sodium in Foods | 1.0 | January 14, 2018 | 0 | 1 | 0 | 3 | 2.5 | 13.60 | 20.10 |
| 7. Zero & Low Sodium Foods | 2.0 | November 5, 2017 | 0 | 1 | 0 | 2 | 8.0 | 13.80 | 24.80 |
| 8. Renal System | 1.0 | March 30, 2015 | 0 | 0 | 1 | 2 | 1.5 | 12.77 | 17.27 |
| 9. Kidney Health Guides | 1.2 | August 9, 2018 | 0 | 0 | 1 | 3 | 15.0 | 14.93 | 33.93 |
| 10. Pukono | 1.6 | July 11, 2017 | 0 | 1 | 1 | 4 | 20.0 | 16.59 | 42.59 |
| 11. RENAL TRKRR | 2.0 | May 5, 2012 | 0 | 1 | 1 | 3 | 6.0 | 11.19 | 22.19 |
| 12. Low Sodium Diet | 1.1.2 | June 19, 2018 | 0 | 0 | 1 | 3 | 12.0 | 13.54 | 29.54 |
| 13. Renal Care Compass - Living  with Dialysis | 1.0 | September 13, 2018 | 0 | 1 | 1 | 7 | 27.0 | 16.42 | 52.42 |
| 14. Aqualert:Drink Water Tracker  & Reminder Google Fit | 7.69 | August 27, 2018 | 1 | 0 | 0 | 5 | 6.0 | 16.25 | 28.25 |
| 15. Mikidney | 1.1 | August 9, 2018 | 0 | 0 | 0 | 6 | 22.5 | 17.29 | 45.79 |
| 16. My Food Coach | 1.2.3 | March 2, 2016 | 0 | 1 | 1 | 5 | 32.0 | 14.82 | 53.82 |
| **Apple iOS-Based** | | | | | | | | | |
| 17. Kidney Diet Food List for Diet | 1.1 | March 30, 2018 | 0 | 0 | 0 | 3 | 0.0 | 9.88 | 12.88 |
| 18. Low Phosphorus Food | 1.2.8 | 2018 | 0 | 1 | 1 | 4 | 24.0 | 13.58 | 43.58 |
| 19. Low Sodium Recipe Plus + | 6.0 | December 3, 2018 | 0 | 1 | 0 | 1 | 0.0 | 13.15 | 15.15 |
| 20. Low Potassium Recipe | 6.0 | June 4, 2016 | 0 | 1 | 0 | 2 | 0.0 | 12.63 | 15.63 |
| 21. Potassium Counter and Tracker  for Healthy | 1.12 | 2017 | 0 | 0 | 0 | 3 | 12.5 | 13.52 | 29.02 |
| 22.Healthy Kidneys Grocery List | 2.0 | 2018 | 0 | 0 | 0 | 1 | 0.0 | 9.28 | 10.28 |

Multimedia Appendix 3. The Scores of Evaluated Dialysis Diet Apps (N=22)
