# Supplementary material for: Limitations of Existing Dialysis Diet Apps in Promoting User Engagement and Patient Self-Management: Quantitative Content Analysis Study
Source: JMIR Mhealth Uhealth. 2020 Jun 1;8(6):e13808. doi: 10.2196/13808 (PMC7296424; doi:10.2196/13808)
Supplement: Multimedia Appendix 4 [file mhealth_v8i6e13808_app4.docx]

| **Dialysis Diet Apps** | **Features** | | | | | | | | | | | | | |
| --- | --- | --- | --- | --- | --- | --- | --- | --- | --- | --- | --- | --- | --- | --- |
|  | **Export of data** | **Gamification** | **General education** | **Plan or orders** | **Reminder** | **Community forum** | **Social media** | **Address symptoms** | **Tailored education** | **Tracker** | **Cost (free)** | **Usability** | **Total point** |  |
| **Android-Based** | | | | | | | | | | | | | |  |
| 1. Phosphorus Foods Diet Guide | **-** | **-** | **✓** | **-** | **-** | **-** | **-** | **✓** | **-** | **-** | **✓** | **✓** | 4 |  |
| 2. Renal Disease Kidney Diet Tips  Symptoms & Foods | **-** | **-** | **✓** | **-** | **-** | **-** | **-** | **✓** | **✓** | **-** | **✓** | **✓** | 5 |  |
| 3. Prevent Kidney Disease | **-** | **-** | **✓** | **-** | **-** | **-** | **-** | **✓** | **-** | **-** | **✓** | **✓** | 4 |  |
| 4. CKD (Chronic Kidney Disease) | **-** | **-** | **✓** | **-** | **-** | **-** | **-** | **-** | **-** | **-** | **-** | **-** | 1 |  |
| 5. Kidney Friend | **✓** | **-** | **✓** | **-** | **-** | **-** | **-** | **✓** | **-** | **-** | **✓** | **✓** | 5 |  |
| 6. Sodium in Foods | **-** | **-** | **✓** | **-** | **-** | **-** | **-** | **-** | **-** | **-** | **✓** | **✓** | 3 |  |
| 7. Zero & Low Sodium Foods | **-** | **-** | **✓** | **-** | **-** | **-** | **-** | **-** | **-** | **-** | **✓** | **-** | 2 |  |
| 8. Renal System | **-** | **-** | **✓** | **-** | **-** | **-** | **-** | **-** | **-** | **-** | **✓** | **-** | 2 |  |
| 9. Kidney Health Guides | **-** | **-** | **✓** | **-** | **-** | **-** | **-** | **✓** | **-** | **-** | **✓** | **-** | 3 |  |
| 10. Pukono | **✓** | **-** | **✓** | **-** | **-** | **-** | **-** | **-** | **-** | **-** | **✓** | **✓** | 4 |  |
| 11. RENAL TRKRR | **✓** | **-** | **-** | **-** | **✓** | **-** | **-** | **-** | **-** | **✓** | **-** | **-** | 3 |  |
| 12. Low Sodium Diet | **-** | **-** | **✓** | **✓** | **-** | **-** | **-** | **-** | **-** | **-** | **✓** | **-** | 3 |  |
| 13. Renal Care Compass - Living  with Dialysis | **✓** | **-** | **✓** | **✓** | **-** | **-** | **-** | **✓** | **-** | **✓** | **✓** | **✓** | 7 |  |
| 14. Aqualert:Drink Water Tracker  & Reminder Google Fit | **-** | **✓** | **-** | **-** | **✓** | **-** | **-** | **-** | **-** | **✓** | **✓** | **✓** | 5 |  |
| 15. Mikidney | **-** | **-** | **✓** | **✓** | **✓** | **-** | **-** | **✓** | **-** | **-** | **✓** | **✓** | 6 |  |
| 16. My Food Coach | **✓** | **-** | **✓** | **✓** | **-** | **-** | **-** | **✓** | **-** | **-** | **✓** | **-** | 5 |  |
| **Apple iOS-Based** | | | | | | | | | | | | | |  |
| 17. Kidney Diet Food List for Diet | **✓** | **-** | **-** | **-** | **✓** | **-** | **-** | **-** | **-** | **-** | **-** | **✓** | 3 |  |
| 18. Low Phosphorus Food | **-** | **-** | **✓** | **-** | **-** | **-** | **-** | **✓** | **-** | **-** | **✓** | **✓** | 4 |  |
| 19. Low Sodium Recipe Plus + | **✓** | **-** | **-** | **-** | **-** | **-** | **-** | **-** | **-** | **-** | **-** | **-** | 1 |  |
| 20. Low Potassium Recipe | **✓** | **-** | **-** | **-** | **-** | **-** | **-** | **-** | **-** | **-** | **-** | **✓** | 2 |  |
| 21. Potassium Counter and Tracker  for Healthy | **-** | **-** | **✓** | **-** | **-** | **-** | **-** | **-** | **-** | **✓** | **-** | **✓** | 3 |  |
| 22.Healthy Kidneys Grocery List | **✓** | **-** | **-** | **-** | **-** | **-** | **-** | **-** | **-** | **-** | **-** | **-** | 1 |  |
| **Frequency (%)** | 9 (41) | 1 (5) | 16 (73) | 4 (18) | 4 (18) | 0 (0) | 0 (0) | 9 (41) | 1 (5) | 4 (18) | 15 (68) | 13(59) |  |  |

Multimedia Appendix 4: The presence of valuable features in evaluated dialysis diet apps from Google Play and the Apple App Store (N=22)
