# Supplementary material for: Limitations of Existing Dialysis Diet Apps in Promoting User Engagement and Patient Self-Management: Quantitative Content Analysis Study
Source: JMIR Mhealth Uhealth. 2020 Jun 1;8(6):e13808. doi: 10.2196/13808 (PMC7296424; doi:10.2196/13808)
Supplement: Multimedia Appendix 5 [file mhealth_v8i6e13808_app5.docx]

Multimedia Appendix 5: The presence of health-behavior theory constructs in evaluated renal diet apps for dialysis from Google Play and the Apple App Store (N=22)

| **Renal Diet Apps** | **Health Behavior Theory Constructs** | | | | | | | | | | | | |
| --- | --- | --- | --- | --- | --- | --- | --- | --- | --- | --- | --- | --- | --- |
|  | **Knowledge** | **Perceived benefits** | **Perceived barrier** | **Perceived risks** | **Self-efficacy** | **Social norms** | **Self-monitoring** | **Goal setting** | **Stimulus control** | **Self-reward** | **Social support** | **Vicarious learning** | **Total point** |
| **Android Based** | | | | | | | | | | | | | |
| 1. Phosphorus Foods Diet Guide | **✓** | **✓** | **-** | **✓** | **✓** | **-** | **✓** | **✓** | **✓** | **-** | **-** | **-** | 7 |
| 2. Renal Disease Kidney Diet Tips  Symptoms & Foods | **✓** | **✓** | **-** | **✓** | **✓** | **-** | **-** | **✓** | **✓** | **✓** | **✓** | **✓** | 9 |
| 3. Prevent Kidney Disease | **✓** | **✓** | **✓** | **✓** | **✓** | **✓** | **-** | **✓** | **✓** | **-** | **-** | **-** | 8 |
| 4. CKD (Chronic Kidney Disease) | **✓** | **-** | **-** | **-** | **-** | **-** | **-** | **-** | **-** | **-** | **-** | **-** | 1 |
| 5. Kidney Friend | **✓** | **✓** | **-** | **✓** | **✓** | **✓** | **-** | **✓** | **✓** | **-** | **-** | **-** | 7 |
| 6. Sodium in Foods | **✓** | **-** | **-** | **-** | **-** | **-** | **-** | **✓** | **-** | **-** | **-** | **-** | 2 |
| 7. Zero & Low Sodium Foods | **✓** | **-** | **-** | **-** | **✓** | **-** | **✓** | **✓** | **-** | **-** | **-** | **-** | 4 |
| 8. Renal System | **✓** | **-** | **-** | **✓** | **-** | **-** | **-** | **-** | **-** | **-** | **-** | **-** | 2 |
| 9. Kidney Health Guides | **✓** | **✓** | **-** | **✓** | **✓** | **-** | **-** | **✓** | **✓** | **-** | **-** | **-** | 6 |
| 10. Pukono | **✓** | **-** | **-** | **-** | **✓** | **-** | **✓** | **✓** | **✓** | **-** | **-** | **-** | 5 |
| 11. RENAL TRKRR | **-** | **-** | **-** | **-** | **-** | **-** | **✓** | **✓** | **-** | **-** | **-** | **-** | 2 |
| 12. Low Sodium Diet | **✓** | **-** | **-** | **-** | **✓** | **-** | **-** | **✓** | **-** | **-** | **-** | **-** | 3 |
| 13. Renal Care Compass - Living  with Dialysis | **✓** | **✓** | **✓** | **✓** | **✓** | **-** | **✓** | **✓** | **✓** | **-** | **-** | **-** | 8 |
| 14. Aqualert:Drink Water Tracker  & Reminder Google Fit | **✓** | **✓** | **-** | **-** | **✓** | **-** | **✓** | **✓** | **-** | **✓** | **-** | **-** | 6 |
| 15. Mikidney | **✓** | **✓** | **-** | **✓** | **✓** | **-** | **✓** | **-** | **✓** | **-** | **-** | **-** | 6 |
| 16. My Food Coach | **✓** | **✓** | **-** | **✓** | **✓** | **✓** | **✓** | **✓** | **✓** | **-** | **-** | **-** | 8 |
| **Apple iOS Based** | | | | | | | | | | | | | |
| 17. Kidney Diet Food List for Diet | **-** | **-** | **-** | **-** | **-** | **-** | **-** | **-** | **-** | **-** | **-** | **-** | 0 |
| 18. Low Phosphorus Food | **✓** | **✓** | **-** | **✓** | **✓** | **-** | **✓** | **✓** | **✓** | **-** | **-** | **-** | 7 |
| 19. Low Sodium Recipe Plus + | **-** | **-** | **-** | **-** | **-** | **-** | **-** | **-** | **-** | **-** | **-** | **-** | 0 |
| 20. Low Potassium Recipe | **-** | **-** | **-** | **-** | **-** | **-** | **-** | **-** | **-** | **-** | **-** | **-** | 0 |
| 21. Potassium Counter and Tracker  for Healthy | **✓** | **✓** | **-** | **-** | **-** | **-** | **✓** | **✓** | **✓** | **-** | **-** | **-** | 5 |
| 22.Healthy Kidneys Grocery List | **-** | **-** | **-** | **-** | **-** | **-** | **-** | **-** | **-** | **-** | **-** | **-** | 0 |
| **Frequency (%)** | 17 (77) | 11 (50) | 2 (9) | 10 (46) | 13(59) | 3(14) | 10 (46) | 15(68) | 11 (50) | 2 (9) | 1 (5) | 1 (5) |  |
